# Prioritizing Conservation of Ungulate Calving Resources in Multiple-Use Landscapes

**DOI:** 10.1371/journal.pone.0014597

**Published:** 2011-01-26

**Authors:** Matthew R. Dzialak, Seth M. Harju, Robert G. Osborn, John J. Wondzell, Larry D. Hayden-Wing, Jeffrey B. Winstead, Stephen L. Webb

**Affiliations:** Hayden-Wing Associates, LLC, Natural Resource Consultants, Laramie, Wyoming, United States of America; University of Pretoria, South Africa

## Abstract

**Background:**

Conserving animal populations in places where human activity is increasing is an ongoing challenge in many parts of the world. We investigated how human activity interacted with maternal status and individual variation in behavior to affect reliability of spatially-explicit models intended to guide conservation of critical ungulate calving resources. We studied Rocky Mountain elk (*Cervus elaphus*) that occupy a region where 2900 natural gas wells have been drilled.

**Methodology/Principal Findings:**

We present novel applications of generalized additive modeling to predict maternal status based on movement, and of random-effects resource selection models to provide population and individual-based inference on the effects of maternal status and human activity. We used a 2×2 factorial design (treatment vs. control) that included elk that were either parturient or non-parturient and in areas either with or without industrial development. Generalized additive models predicted maternal status (parturiency) correctly 93% of the time based on movement. Human activity played a larger role than maternal status in shaping resource use; elk showed strong spatiotemporal patterns of selection or avoidance and marked individual variation in developed areas, but no such pattern in undeveloped areas. This difference had direct consequences for landscape-level conservation planning. When relative probability of use was calculated across the study area, there was disparity throughout 72–88% of the landscape in terms of where conservation intervention should be prioritized depending on whether models were based on behavior in developed areas or undeveloped areas. Model validation showed that models based on behavior in developed areas had poor predictive accuracy, whereas the model based on behavior in undeveloped areas had high predictive accuracy.

**Conclusions/Significance:**

By directly testing for differences between developed and undeveloped areas, and by modeling resource selection in a random-effects framework that provided individual-based inference, we conclude that: 1) amplified selection or avoidance behavior and individual variation, as responses to increasing human activity, complicate conservation planning in multiple-use landscapes, and 2) resource selection behavior in places where human activity is predictable or less dynamic may provide a more reliable basis from which to prioritize conservation action.

## Introduction

Identifying resources associated with critical life-history phases in ungulates is a conservation priority. Winter range, parturition areas, and migration routes are important seasonal habitats in North America that provide resources necessary for survival and reproduction such as high-quality forage, reduced exposure to inclement conditions, and reduced risk of predation [1], [2]. In the Intermountain West USA, these habitats have become increasingly fragmented. Here, energy development is of broad conservation interest because its prominence has increased in recent decades along with concern about its potential impact on wildlife and their habitats [3]. Understanding how human activity such as resource extraction interacts with wildlife, and developing tools to guide conservation planning in areas where human activity is widespread or increasing are ongoing challenges in conservation science. In this paper, we investigated interactions between human activity associated with energy development and resource selection by female Rocky Mountain elk (*Cervus elaphus*) during calving season with the larger goal of informing conservation planning for ungulates in places where human activity is widespread or increasing. We asked: 1) what are the relative influences of maternal status (parturiency) and human activity on resource selection, 2) to what extent does behavior vary among individuals, 3) how does individual variation interact with human activity, and 4) how can our findings be applied in conservation planning and decision making? First, we describe a novel application of generalized additive models for designating maternal status using movement data. Model-based methods to designate maternal status were necessary because data bearing directly on maternal status were unavailable for most elk. Second, we estimated group-dependent random-effects resource selection functions (RSFs) to identify how resource selection patterns differed relative to maternal status and human activity, and how behavior varied among individuals. We applied results by developing and validating group-dependent predictive maps of critical calving resources, and quantifying discrepancies among maps relative to predictive accuracy.

A key approach in studying wildlife-human interaction is resource selection modeling [4]. Resource selection is a fundamental ecological process that structures animal movement and distribution [5]. The choices animals make as they move throughout the landscape reflect trade-offs between selecting resources that meet their needs for survival and reproduction, and minimizing perceived risk of harm – such risk often is a function of interaction with predators or humans [6], [7], [8]. A set of analytical methods used with increasing frequency for investigating resource selection in animals is the estimation of RSFs [9]. RSFs describe the relative probability of occurrence of animals as a function of behavioral responses to features of the environment. The probability of occurrence is described as relative because RSFs are estimated in a use-versus-availability framework in which selection is quantified relative to available but presumed non-used features. Environmental features can include a wide range of variables such as vegetation, terrain, group/herd size, risk of predation, or human-modifications of the landscape [10], [11]. RSFs have strong application in conservation planning where wildlife-human interaction is a concern; specifically, in establishing a spatially explicit basis from which to prioritize conservation action such as reclamation, mitigation, or minimizing human activity in particular habitats.

Areas associated with parturition are important because female ungulates make resource-related choices that affect offspring development during gestation and provisioning of recently born calves that are susceptible to malnutrition and predation [12], [13]. While there is little evidence of consistent parturition site fidelity in many ungulates, strong fidelity among females to seasonal ranges, particularly around calving time, has been demonstrated [2], [14]. New light has been shed on the adaptive significance of resource selection during the period that encompasses reproductive activity in many vertebrates through the study of maternal effects – developmental mechanisms by which parents translate their environmental experience into adaptive variation in their offspring [15], [16], [17]. The adaptive significance of resource selection during reproductive periods suggests that conservation strategies designed around ungulate parturition areas might be most reliable when based on parturient females rather than samples including both parturient and non-parturient individuals.

Another key feature of animal ecology that warrants further attention as part of conservation planning is variation in behavior among individuals. Individual variation is widespread and well-known in many animal species [18] and can reflect long-term selection for a given trait or learned behavior [19], [20]. Fitness is influenced by the choices individuals make in terms of resource selection because each resource type has particular costs and benefits to the individual [21]. In risky or rapidly changing environments such as those in which human activity is increasing, optimal behavioral strategies may vary among individuals on a situation-specific basis [22] making it difficult to generalize behavior across the population and thus effectively guide conservation planning.

## Methods

### Study area

**Figure 1:**
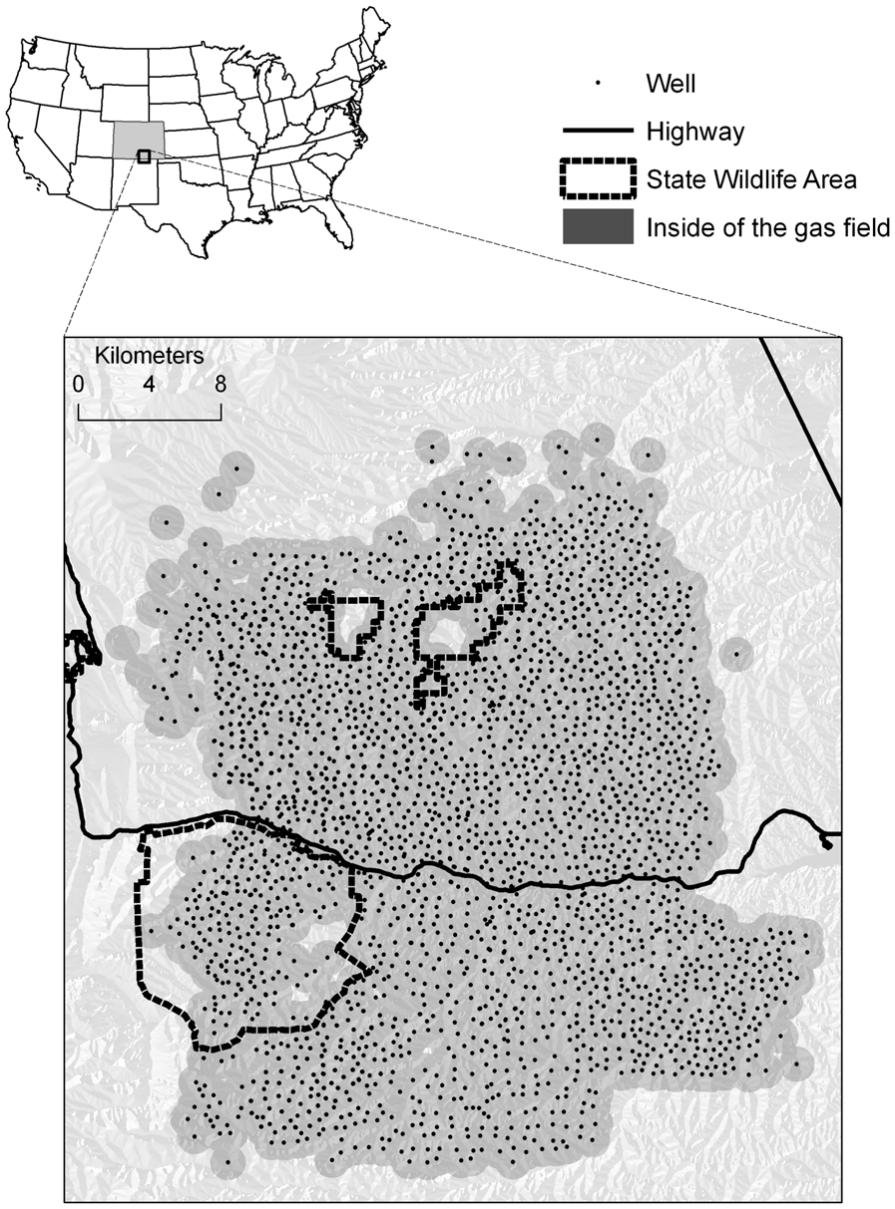
The Raton Basin gas field and adjacent areas in south-central Colorado, USA. Each natural gas well is encircled by a 1-km buffer (shaded region). We designated elk locations occurring within the buffered region to be “inside of the gas field” whereas locations adjacent but external to the buffered region were designated “outside of the gas field”.

The 1845 km^2^ study area encompassed northern portions of the Raton basin in south-central Colorado, USA. Topography is rugged with steep slopes, rocky outcrops, ridges, and valleys ranging in elevation from 2000–3000 m. Mean annual precipitation is about 40–53 cm depending on elevation [23]. Vegetation includes conifer forest, montane shrub, and grassland. Dominant species include ponderosa pine (*Pinus ponderosa*), one-seed juniper (*Juniperus monosperma*), two-needle pinyon (*Pinus edulis*), Gambel oak (*Quercus gambelii*) which commonly forms shrub-thickets on southern aspects, antelope bitterbrush (*Purshia tridentata*), skunkbush sumac (*Rhus trilobata*), and willow (*Salix* spp.) in riparian areas. Predators of elk (including neonates) include black bear (*Ursus americanus*), mountain lion (*Felis concolor*), and coyote (*Canis latrans*); no wolf (*Canis lupus*) pack occurred in the study area. The study area encompassed historic and ongoing energy development. Bituminous coal mining was a dominant land use during 1873–1970. Coal-bed methane development was initiated in Raton basin in 1982 and accelerated in the late 1990s [24], [25]. In 2009, there were about 2900 wells associated with methane development in the Basin (Figure 1).

### Capturing elk

In February and March 2006–2009, helicopter net-gunning was used to capture yearling (1.5 years) and adult (≥2.5 years) female elk throughout and adjacent to the gas field. Elk were fitted with Global Positioning Systems (GPS) collars (TGW-3590, Telonics, Inc., Mesa, AZ 85204) configured with store-on-board and Very High Frequency (VHF) beacon options. Twenty-five female elk were fitted with GPS collars in 2006, 40 in 2007, 50 in 2008, and 50 in 2009. GPS collars attempted to record location information every 3 h resulting in a maximum of 8 locations/elk/day. Age of elk was estimated based on dental eruption and wear patterns [26]. Blood samples were collected from captured elk in 2008 and 2009 to determine pregnancy, but not from elk captured in previous years. Animal capture and handling protocols were approved by the Colorado Division of Wildlife (Permit #s 06TR1083, 07TR1083, 08TR1083 and 09TR1083A001).

### Grouping Elk Relative to Maternal Status and Human Activity

**Figure 2:**
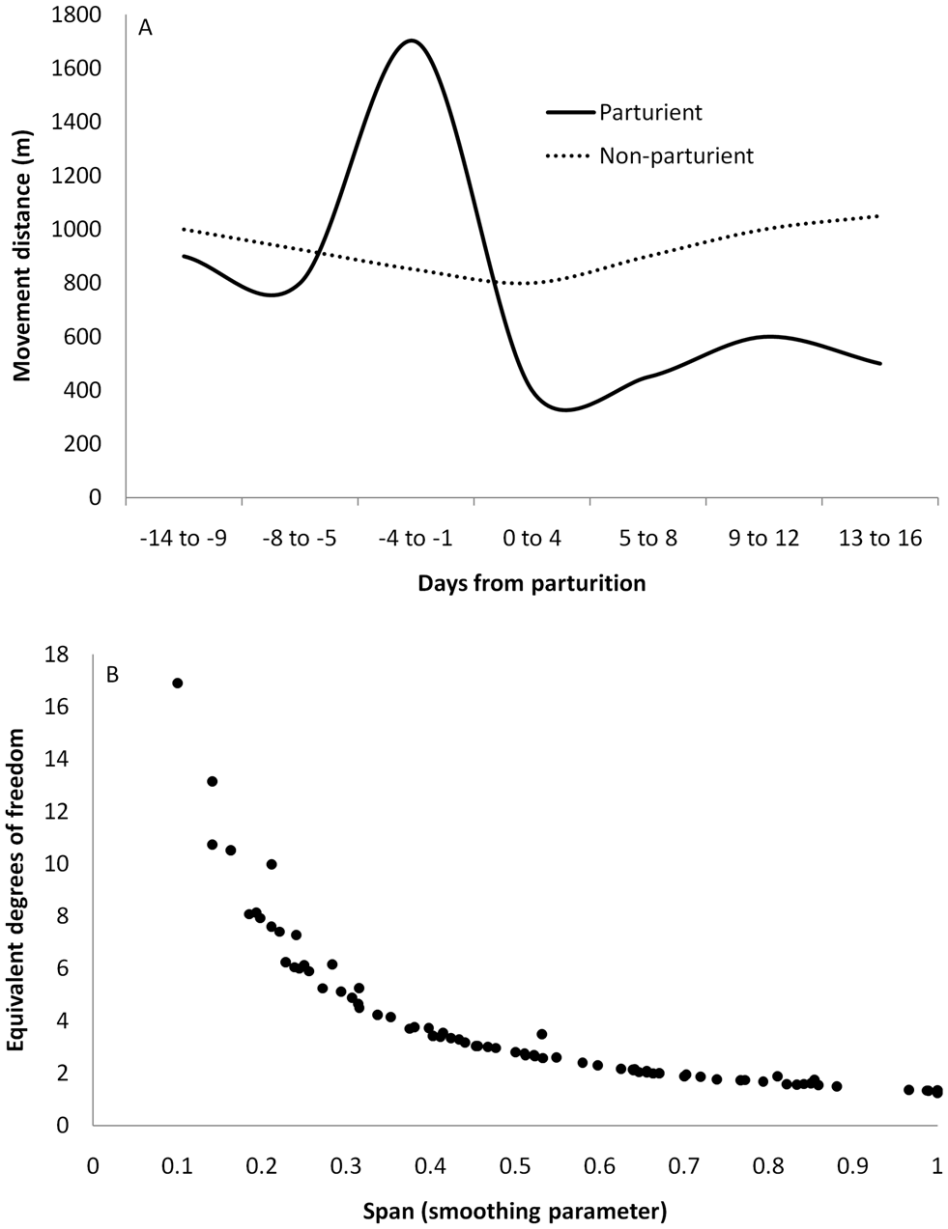
Movement in female ungulates during calving time. Generalized from the literature [34], [35], [36], parturient females often will make long-distance movements associated with pre-parturient “restlessness” within days of parturition and then exhibit reduced movement associated with provisioning the neonate (a). This pattern may provide a quantifiable distinction between parturient and non-parturient females. Also shown (b) is parameterization (equivalent degrees of freedom) as a function of span based on generalized additive models of distance moved between consecutive locations in 103 female Rocky Mountain elk. Loess smoothing and automated calculation of degrees of freedom using the generalized cross validation method were specified.

We predicted maternal status (parturient versus non-parturient) by using generalized additive models (GAMs) to parameterize response curves depicting daily movement of elk during calving season. GAMs are semi-parametric extensions of generalized linear models [27]. The central concept is that the function of a covariate is estimated nonparametrically from the data by means of scatterplot smoothers. The functional form of the relationship between the response and covariate(s) is therefore determined by the data rather than being restricted to a parametric form [28]. Formally, the linear regression model
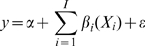
is generalized by modeling *y* as being related to covariates additively by
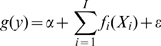
where *g* is the link function and *ε* is a random error term. Functions *f_i_* may be linear or nonparametric functions defined by smoothers such as smoothing splines or locally estimated scatterplot smoothers (loess; also referred to as locally estimated, or weighted, polynomial regression). Smoothers provide a series of data summaries of the response that are specific to regions of the covariates; a well known smoother is the moving average [29]. The amount of smoothing is calibrated by the size of the neighborhood, or percentage of the data points, over which averaging is done; a quantity known as span. A larger span yields a smoother data summary (less curvature) whereas a smaller span yields a less smooth data summary (more curvature). This data summary (*i.e.*, the effective number of parameters of a smoother) is described by the quantity equivalent degrees of freedom (*df*). Span is related inversely to *df* so as span increases *df* decreases [27]. Data that are best described by a straight line (a single parameter comprises the smoother) correspond to a span of 100% and thus 1 *df*. Conversely, data that are best described by gradients or turning points (several or many parameters comprise the smoother) correspond to a smaller span and thus to a larger number of *df* [27], [30]. The analyst may set the span by specifying *df* based on visual examination of the data, or implement generalized cross validation methods in which appropriate *df* are identified automatically given the data. From a GAM perspective, movement of female ungulates during the calving season likely contains information on maternal status. Restlessness and the seeking of solitude characterize imminent parturition [31], [32], [33] and establish a general pattern of increased daily movement pre-partum and decreased movement post-partum relative to barren females (Figure 2a; [34], [35], [36]). We hypothesized that during calving time, movement associated with restlessness and solitude seeking in parturient elk provides a general pattern of complexity that is not observed in non-parturient females, and we predicted that smoothers associated with movement data on parturient elk consistently would be comprised of more parameters and thus more *df* than smoothers associated with non-parturient elk.

We used GAMs to regress distance traveled within a 24 h period (using locations recorded at 1200 h on consecutive days) against date. Date encompassed the 15 May – 1 July calving season [33] in each of the 4 years comprising the study period. We used the generalized cross validation option to assign *df* to response curves depicting daily changes in the distance moved by elk between successive locations. We used PROC GAM in SAS® (SAS Institute, Inc., Cary, North Carolina, USA), specifying automated calculation of *df* using the generalized cross validation method and loess smoothing, to assign *df* to response curves depicting calving season movement patterns in each elk. We established the following prediction: movement described by <*3 df* in GAMs would depict non-parturiency whereas movement described by ≥3 *df* would depict parturiency. This prediction was based on two observations. First, the shape of the response curve depicting relatively simple movement, as in non-parturient individuals, appears to correspond with ≤2 regions that differ in slope, each of which being calibrated over a span of ≥50% of the data (Figure 2a; [34], [35]). Second, by plotting *df* as a function of span, our data show that a span of 50% corresponds to ∼3 *df* (Figure 2b).

We tested the GAM approach using blood samples obtained from elk during capture in 2008 and 2009, and field observation of females and calves in those same years. Blood sera were tested for presence of pregnancy-specific protein-B (PSPB; BioTracking, LLC, Moscow, Idaho, USA; [37]). We conducted field observation from dawn to 0900 and from 1800 until dusk using binoculars and spotting scopes to watch for behavior that suggested a maternal bond between a female and calf. This behavior included nursing and licking bouts, traveling as a female/calf unit, and heightened attentiveness between a female and calf [13], [38]. Females for which PSPB testing indicated pregnancy and for which field observation suggested a strong female-calf bond were designated as parturient. Determining non-parturiency is never definitive; however, we designated females as non-parturient with negative PSPB results and for which field observation was unable to associate the female with a calf [13]. We thus established two groups of elk relative to maternal status by which we analyzed resource selection.

Similarly, we established two groups of elk relative to human activity: elk occupying developed areas versus elk occupying undeveloped areas. Elk locations occurring within 1 km of a gas well were considered to be in the developed area (Figure 1). Human activity was apparent in the areas we called undeveloped including some ranching and residences; however, no industrial development occurred in undeveloped areas and human activity was limited relative to developed areas. Given the short temporal window within which we conducted these analyses, calving season use areas (100% minimum convex polygons) generally occurred wholly within developed areas or wholly within undeveloped areas. Only a small number of elk occupied both areas during the calving season – we discuss these elk separately. We estimated minimum convex polygons because the temporal window of the study was relatively short, calving season use areas comprised a portion of the annual use areas, and we wanted to err on the side of inclusiveness rather than potentially omitting a portion of critical calving range from the analysis. In comparing resource selection between elk occupying developed versus undeveloped areas, a key assumption is that human activity associated with energy development is the primary difference between areas and that other factors to which elk respond were similar between areas. We compared landscape and habitat covariate values between areas to inform this assumption.

### Random-effects Resource Selection Modeling

Modeling variables as random effects can improve our understanding of resource selection, which will enhance the practical application of RSFs in management decisions [4], [39]. Random effects models assume that sample units are drawn at random from a larger population and that the data are structured hierarchically (*i.e.*, within subject responses are more similar than between-subject responses). Mechanistically, assumptions of random-effects models are: (1) the random effects are distributed normally with mean equal to 0 and unknown variance, (2) within-group correlation is constant through time, and (3) the analyst has correctly specified the variance-covariance structure (see [39] for a review of the application of random-effects models in resource selection analysis). In RSF models, it is appropriate to model intercepts and/or covariates as random effects when variance among sample units is of interest, animal response to gradients in a resource is suspected, or population-level inference is of interest. Random intercepts can account for unbalanced data, correlation among observations, and provide improved model fit and parameter estimation [39] (*but see* [11]). Random-effects (or mixed-effects) RSF models provide information on individual behavior, how individuals contribute to population-level observations, and how their responses to a resource may change as a function of its availability – a process known as a functional response [40], [41]. Analytical approaches to model functional responses in resource selection are particularly important when there is a trade-off in selection for a particular resource [4], [40], which would be the case if human activity is perceived as a risk of harm [7].

We incorporated random effects into the use versus availability design [9] in which covariates representing important resources are compared at used and available (but presumed non-used) locations using
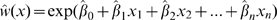
where 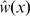 is the relative probability of use as a function of covariates *x_n_* with coefficients 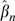 estimated from logistic regression. Availability was defined for each elk by including random locations within 100% minimum convex polygon seasonal use area estimates; the number of random locations generated was 3 times the number of used locations for each elk. We examined resource selection within seasonal use areas (*i.e.*, 3^rd^ order selection). We modeled resource selection separately during day and night because we expected behavior of elk to differ between day and night. We assigned time of day at random to available locations for day versus night comparisons; times assigned to available locations corresponded to times associated with used locations (e.g., every 3 hours on the sampled hour). Day models included the times 0900, 1200, 1500, and 1800 h whereas night models included 0000 and 0300 h. Using a Geographic Information System (GIS; ArcGIS 9.2), we calculated 7 covariates at used and random locations (Table 1). Four of these covariates including cover type, slope, elevation, and habitat edge density were calculated at locations both within and outside of the gas field. Three covariates including road density, distance to a human-built structure, and industrial development footprint (Table 1) were calculated only within the gas field because human activity in areas adjacent but outside of the gas field was less intense and not associated with industrial development. Raster data for cover type were developed from annual aerial photography of the study area, terrain covariates were calculated from a 30-m resolution digital elevation model, and human activity covariates including roads, structures, and industrial development footprint were heads-up-digitized from aerial photography and analyzed as year-specific covariates (Table 1). We used Spatial Analyst in ArcGIS to extract values from raster data for all covariates.

**Table 1:** Covariates used in random-effects resource selection models and their descriptions.

Covariate	Description
Slope	Digital elevation model (DEM) provided at a resolution of 1.52 m and re-sampled for covariate calculation to a resolution of 30 m and measured in degrees. Values for slope calculated at point locations in our study ranged from 0.02 (flat ground) to 41.8 degrees (very steep).
Elevation	DEM re-sampled for covariate calculation to a resolution of 30 m and measured in m. Elevation at sample points in our study ranged from 1918.7–2970.0 m.
Cover type	Raster dataset compiled from 1-foot resolution aerial imagery of the study area using Image Analysis™ and re-sampled to 30 m resolution for covariate calculation. From an elk-centric perspective, habitat in the Raton basin functioned in one of two ways: as security cover or as forage resources. Raster cells were assigned one of two values (binary covariate) representing habitat that functioned to provide cover versus habitat that did not function to provide cover. All tree or oak-thicket dominated habitats were considered security cover, whereas all shrub and grassland dominated habitats were considered non-cover.
Edge density	Density of line features depicting the interface of cover and non-cover habitat calculated for the central grid cell within a 990 m^2^ moving window. Values in our study were 22.2–136.0 km/km^2^.
Road density	Density of line features depicting roads calculated for grid cells at the center of a 990 m^2^ moving window. Road density was calculated as a year-specific variable – that is, new road features were added to the data set as annual aerial imagery became available. Values in our study, calculated within the gas field only, were 0.0–7.6 km/km^2^.
Distance to structure	Linear distance from a sample location to a human-built structure including houses, agricultural facilities, and industrial facilities. Distance to structure was a year-specific variable and was analyzed as natural log transformed distance +0.1 to allow its magnitude to decrease with increasing distance. Values in our study, calculated within the gas field only, were 0.0–5854.9 m.
Industrial development footprint	Area (density) of physically modified ground calculated from aerial photography as a year-specific variable. Physically modified ground primarily reflected industrial development including well pads, pipe yards, pipelines, construction areas, or clearing for facilities development. Industrial development footprint was calculated for the central grid cell within a 990 m^2^ moving window. This covariate excludes physical disturbance associated with roads. Values in our study, calculated only within the gas field, were 0.0–0.62 km^2^/km^2^.

We estimated a three-level random-effects model in which locations *i* = 1…*I* occurred within strata representing individual elk *j* = 1…*J* [39]. Considering a random intercept and random coefficients, the RSF is estimated by
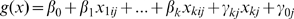
where covariates *k* (*k* = 1…*K*) have values *x*, 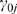 is the random intercept and 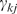 is the random coefficient of *x_k_* for elk *j*, which is the difference in the intercept and coefficient for elk *j* from the mean population-level intercept *β*_0_ and coefficient *β_kj_*, respectively. We estimated models using the GLIMMIX procedure in SAS®. We specified the conditional probability distribution of the data as binomial and used a logit link function. We included ordinal date as a class variable and specified a variance components covariance structure in which random intercepts for ordinal date were nested within each individual to address within-day autocorrelation among locations. R-side, or marginal, random-effects models estimated using GLIMMIX provide conditional coefficient estimates for each individual, that is, estimates for individual animals that are conditional on the distribution of coefficient estimates across all individuals in the population. To estimate marginal (*i.e.*, population-level) coefficients we assumed that conditional coefficients for each elk represented a random sample from a normal distribution with the mean of that distribution representing the population-level effects of covariates on the probability of use [42]. We estimated marginal coefficients using
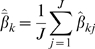
where 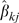 was the estimate of coefficient *k* for elk *j* [43], [44], and we estimated variance using
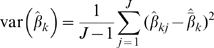



Population-level coefficient estimates 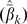 are similar to the average of the conditional estimates because the conditional coefficient estimates are constrained to have means 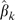 [44]. In all, five groups of elk were available for comparison: 1) all sample elk inside of the gas field, 2) elk predicted to be parturient based on GAMs inside the gas field, 3) field-observed parturient elk inside of the gas field, 4) field-observed non-parturient elk inside of the gas field, and 5) all sample elk outside of the gas field (sample size was too low to develop maternal groups; see Results).

### Mapping Responses

We applied the results from random-effects models to map relative probability of use across the study area which encompassed developed and undeveloped areas. We used marginal coefficients from logistic regression to derive an RSF at a resolution of 30 m using
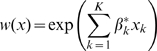
where covariates *k* (*k* = 1…*K*) have values *x* [9]. We were interested in differences in resource selection patterns depending on maternal status and between elk that occupied developed versus undeveloped areas. It is important to note that, although we analyzed an independent group of field-observed parturient elk to facilitate comparison of marginal estimates, these elk were withheld from final RSF development as a validation sample. Thus, we examined differences in how the relative probability of use was assigned throughout the landscape depending on whether this probability was based on models of: 1) all sample elk inside the gas field, 2) elk predicted to be parturient based on GAMs inside the gas field, and 3) all sample elk outside of the gas field (groups 1,2, and 5 above). We conducted this examination using the following methods separately for day and night. We developed annual RSF maps for each of the 3 groups identified above. We estimated quantiles in SAS® (PROC RANK, PROC MEANS) by which pixels comprising the raster surface were partitioned into 5 equal-sized subsets based on pixel value. In GIS we reclassified RSF values based on quantiles establishing 5 ranks of the relative probability of use (1 = low probability, 5 = high probability). We summed within-year maps across all years and ranked relative probability of use as described above yielding 6 multi-year predictive maps (day map and night map for each group) with relative probability of use ranging from 1 (low) to 5 (high). We validated predictive maps using locations from 24 elk that were observed in the field to be parturient (see below); these elk were withheld from final RSF development so they represent an independent validation sample. Locations from these elk were plotted on multi-year predictive maps. We tested whether the number of locations that occurred within each predicted probability of use rank (1–5) differed from expectation using a chi-square test for specified proportions (PROC FREQ, SAS®). To provide a measure of the amount to which a map that validated well differed from a map that validated poorly, we calculated the number of pixels comprising the raster surface that differed between maps in terms of relative probability rank.

## Results

### Capturing Elk and the Environment Inside versus Outside of the Gas Field

We fitted 25 female elk with GPS collars in 2006, 40 in 2007, 50 in 2008, and 50 in 2009. The entire within-year sample was unavailable for these analyses because the analyses spanned a relatively short period during calving (Table 2), and in each year several elk moved from the study area to alpine habitat for parturition. Inadequate sample size (*i.e.*, ≤2 elk comprising maternal groups) in undeveloped areas restricted a more comprehensive assessment. Differences in elevation, slope, edge density, and cover type between developed and undeveloped areas were minimal. Based on 25,290 GIS-generated random sample points (12,645 inside and 12,645 outside of the gas field, respectively), 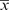 ± SD values inside versus outside of the gas field respectively were 2,347.7 ± 146.4 m and 2,382.6 ± 243.4 m for elevation, 10.6 ± 6.2 degrees and 9.4 ± 7.0 degrees for slope, and 82.0 ± 10.2 km/km^2^ and 77.5 ± 12.7 km/km^2^ for edge density. The proportion of sample points that occurred within security habitat was 0.84 and 0.80 inside versus outside of the gas field, respectively. Topographic covariates (elevation and slope) were not ground truthed; however, vegetation attributes were ground-truthed based on field-established polygons (*n* = 1177) of known vegetation type.

**Table 2:** Resource selection by Rocky Mountain elk during calving time; sample size and number of locations used in analyses.

		#Elk		#Locations	
Year	Group^a^	Day	Night	Day	Night
2006	Inside – all sample elk	11	10	1,759	879
	Inside – GAM-predicted parturient	5	5	917	458
	Outside – all sample elk	7	7	122	61
2007	Inside – all sample elk	26	24	3,281	1,641
	Inside – GAM-predicted parturient	9	9	1,174	587
	Outside – all sample elk	16	14	900	451
2008	Inside – all sample elk	19	19	2,056	1,455
	Inside – GAM-predicted parturient	7	7	776	438
	Inside – field-observed parturient	12	12	1,598	802
	Inside – field-observed non-parturient	5	5	425	227
	Outside – all sample elk	19	16	417	230
2009	Inside – all sample elk	15	15	2,735	1,482
	Inside – GAM-predicted parturient	8	8	1,381	757
	Inside – field-observed parturient	12	12	1,976	1,062
	Inside – field-observed non-parturient	5	5	685	496
	Outside – all sample elk	16	14	411	215

a Inside refers to areas within the gas field and outside refers to areas adjacent but external to the gas field. GAM-predicted parturient refers to female elk that we predicted to be parturient based on results of generalized additive modeling (GAM) of movement data. Field-observed refers to elk for which we observed behavior in the field to assess parturition status.

Total sample fitted with Global Positioning Systems collars was 25, 40, 50, and 50 in 2006, 2007, 2008, and 2009, respectively. Within-year deviation from the available sample arises from 3 sources: 1) some collared elk moved out of the study area during calving, 2) the GAM-predicted sample was nested within the total sample, and 3) some individuals occupied areas both inside and outside of the gas field.

### Generalized Additive Modeling to Designate Maternal Status

We applied results from PSPB and field observation of female elk in 2008 and 2009 to test the GAM approach, in which movement patterns parameterized by <*3df* were predicted to depict non-parturiency whereas movement patterns parameterized by ≥3 *df* were predicted to depict parturiency. Based on PSPB results and 788 hours of field observation, we assigned maternal status to 34 elk; 24 were identified as parturient and 10 as non-parturient. Movement during calving time was highly variable, especially among parturient elk, with parameterization ranging from 1.24–16.9 *df*. However, among elk observed to be parturient in the field, 93% (13/14) were parameterized by ≥3 *df* (Table 3).

**Table 3:** Parameterizing movement using generalized additive models.

Year/Group	*df*<2^a^	2<*df*<3	*df≥*3
2008 Field-observed parturient	2	1	9
2009 Field-observed parturient	5	3	4
2008 Field-observed non-parturient	2	2	1
2009 Field-observed non-parturient	3	2	0

a As part of generalized additive modeling we specified the generalized cross validation option to assign parameters (*df*) to polynomials depicting daily changes in the distance moved by elk between successive locations.

Maternal status was assigned to 34 female Rocky Mountain elk (10 non-parturient and 24 parturient) using information from blood samples (pregnancy-specific protein-B) and field observation (788 h). We assessed how generalized additive models of location data parameterized movement, and how parameterization corresponded with calf status as determined in the field. Results show that among 14 elk for which movement was parameterized by ≥3 *df*, 13 were observed in the field to be with calf.

### Random-effects Resource Selection Modeling – Marginal Inference

**Figure 3:**
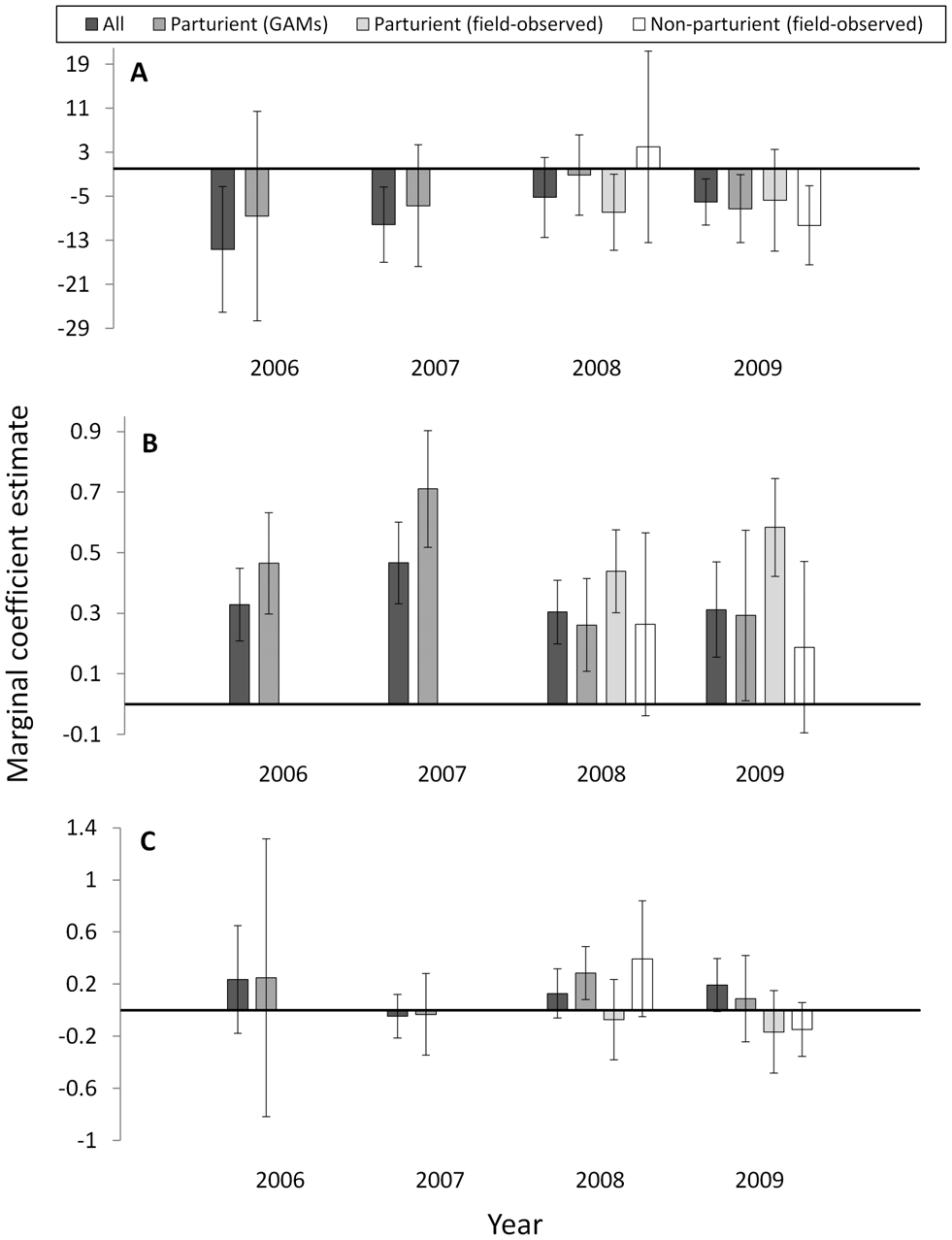
Group-dependent population-level responses: inside of the gas field. Marginal coefficient estimates ± 95% CL of selection for human disturbance (a), security cover (b), and road density (c) by female elk during 2006-2009 in Raton Basin, Colorado, USA. Day time results are displayed among maternal statuses for elk inside of the gas field.

We found little evidence of lack of fit or overdispersion in RSF models; generalized chi-square/degrees of freedom was 0.79–0.92 for night time models and 0.87–0.97 for day time models. The most notable response by elk inside of the gas field was strong avoidance of the industrial development footprint during the day among all groups (at the population-level; Figure 3a). Other notable behavior in field-observed parturient elk included strong selection for cover and avoidance of high road density during the day relative to other groups (Figure 3b,c). While field-observed parturient elk showed some day-time preference for flatter areas and lower elevation relative to the larger sample, differences among maternal groups in selection for these and other resources were small, inconsistent, or not apparent.

**Figure 4:**
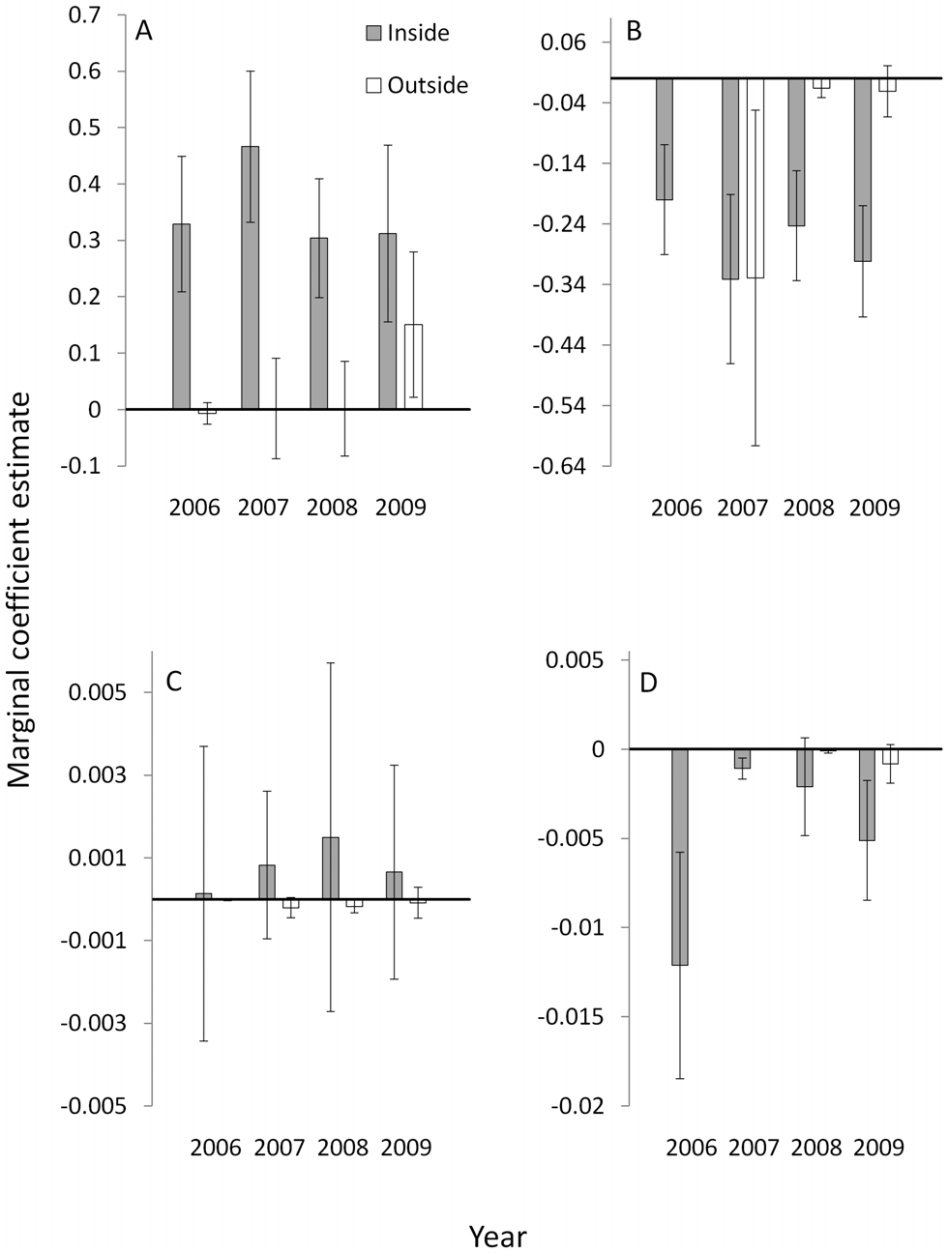
Population-level responses: inside versus outside of the gas field. Marginal coefficient estimates ±95% CL of day-time (a) and night-time (b) selection for security cover, and day-time (c) and night-time (d) selection for elevation by female elk during 2006-2009 in Raton Basin, Colorado, USA. Inside refers to elk occupying an active gas field whereas outside refers to elk occupying adjacent undeveloped areas; maternal status is not considered (*i.e.*, all sample elk are grouped depending on whether they occurred inside versus outside of the gas field).

We estimated selection for 4 landscape/habitat covariates in both developed and undeveloped areas including security cover, slope, elevation, and habitat edge density. Considering population-level coefficient estimates, selection for resources that functioned to provide security cover differed markedly between elk that occupied developed areas versus undeveloped areas (Figure 4). Elk inside of the gas field consistently showed strong selection for security cover during the day (Figure 4a) and strong selection for non-cover or forage habitats during the night (Figure 4b) throughout the study period whereas elk occupying areas outside of the gas field generally selected randomly for security cover with coefficient estimates near zero. Another difference between elk inside versus outside of the gas field involved selection for elevation. Elk outside of the gas field generally selected randomly for elevation with coefficient estimates always near zero. Elk inside of the gas field generally selected for higher elevation during the day (Figure 4c; however, 95% CL overlapped zero), and for lower elevation at night (Figure 4d). Diurnal patterns in selection for slope and edge density were apparent but differences in selection for these features between elk inside versus outside of the gas field generally were small (Table 4).

**Table 4:** Marginal coefficient estimates (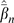
[SE]) for two-level random-effects models of resource selection by Rocky Mountain elk during calving time (15 May - 1 July), 2006–2009.

	Day		Night	
Effect/Year	Inside	Outside	Inside	Outside
Slope 2006	−0.001 (0.002)	−0.003 (0.034)	−0.064 (0.013)	-
Slope 2007	0.011 (0.006)	0.005 (0.013)	−0.030 (0.010)	−0.040 (0.016)
Slope 2008	−0.003 (0.007)	0.003 (0.003)	−0.035 (0.007)	−0.045 (0.008)
Slope 2009	0.012 (0.005)	0.024 (0.014)	−0.023 (0.011)	−0.027 (0.013)
Edge Density 2006	0.015 (0.016)	−0.022 (0.004)	−0.007 (0.021)	-
Edge Density 2007	0.001 (0.013)	−0.003 (0.012)	0.006 (0.011)	−0.013 (0.004)
Edge Density 2008	−0.026 (0.009)	−0.008 (0.003)	−0.026 (0.008)	−0.010 (0.002)
Edge Density 2009	−0.027 (0.009)	−0.006 (0.010)	−0.036 (0.012)	−0.044 (0.021)

- Model failed to converge.

### Random-effects Resource Selection Modeling – Conditional Inference

**Figure 5:**
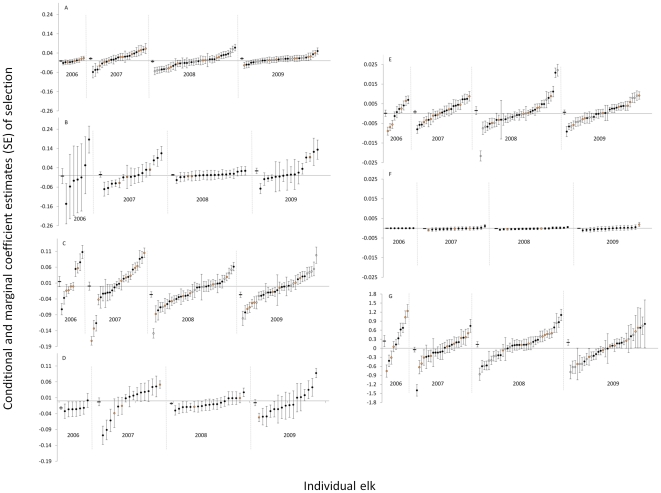
Population-level and individual responses. Marginal (dash) and conditional (circles) coefficient estimates (SE) of day-time selection for slope (a, b) edge density (c, d), elevation (e, f), and road density (g) by female elk during 2006-2009 in Raton basin, Colorado, USA. Results are displayed by year for elk that occupied developed (panels a, c, e, and g) and undeveloped (panels b, d, and f) areas. Orange depicts elk that were predicted to be parturient using generalized additive modeling; open circles (○) indicate conditional estimates for field-observed parturient elk in 2008 and 2009.

Conditional estimates revealed a complex association between human activity and individual variation in response to environmental and anthropogenic features (Figure 5). Elk inside the gas field showed greater heterogeneity among individuals relative to elk outside of the gas field in their responses to edge density and elevation. Selection for slope (Figure 5a,b) and edge density (Figure 5c, d) was estimated more precisely among elk inside of the gas field; however, elk outside the gas field selected (randomly) for elevation with high precision (Figure 5e, f). Also apparent inside the gas field but not outside was a temporal trend of increasing avoidance of high edge density (Figure 5c). Within-year marginal estimates of day time selection for road density were positive (*i.e.*, elk selected for higher road density) in 3 of 4 years. However, conditional estimates revealed that, across all years, only 37% of sampled elk selected for relatively high road density; 32% selected neither for nor against high road density, and 31% avoided high road density (Figure 5g).

**Figure 6:**
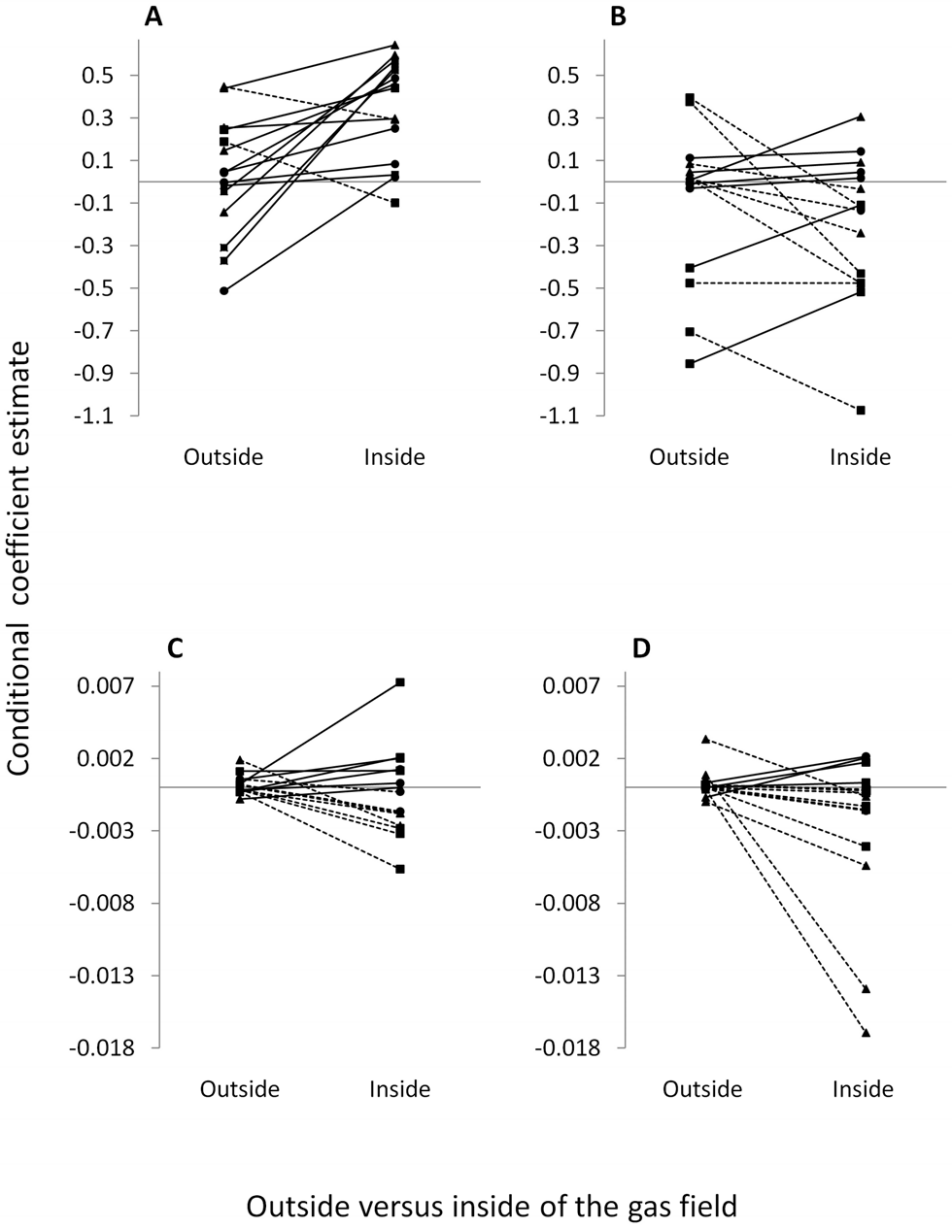
Movement from within the gas field to areas adjacent to the gas field. A subsample of female elk (*n* = 15) occupied areas within the gas field as well as areas outside but adjacent to the gas field. This figure shows differences in day-time (a) and night-time (b) selection for habitat that provides security cover, and day-time (c) and night-time (d) selection for elevation among these elk depending on whether they were inside versus outside of the gas field. Symbols represent conditional coefficient estimates (y-axes) for each elk in 2007 (squares; *n* = 6), 2008 (circles; *n* = 4), and 2009 (triangles; *n* = 5); lines show how selection changed within each elk depending on whether it was inside versus outside of the gas field. Solid lines depict larger selection coefficients inside the gas field whereas dashed lines depict larger coefficients outside of the gas field. Coefficients are informed by 36-293 location per elk inside the gas field, and 20-248 locations per elk outside the gas field.

Seasonal use areas of 15 elk overlapped both developed and undeveloped areas. Individuals showed notable behavioral differences, including diurnal variation, relative to selection for security cover and elevation depending on whether they were in developed versus undeveloped areas (Figure 6). During the day, 13 of 15 elk showed stronger selection for security cover when inside the gas field, but at night this pattern broke down with no apparent consistent behavioral response. Selection for elevation was variable inside the gas field during day and night with some elk showing relatively strong selection for higher or lower elevations. When elk were outside of the gas field they generally used elevation at random (Figure 6).

### Functional Responses

**Figure 7:**
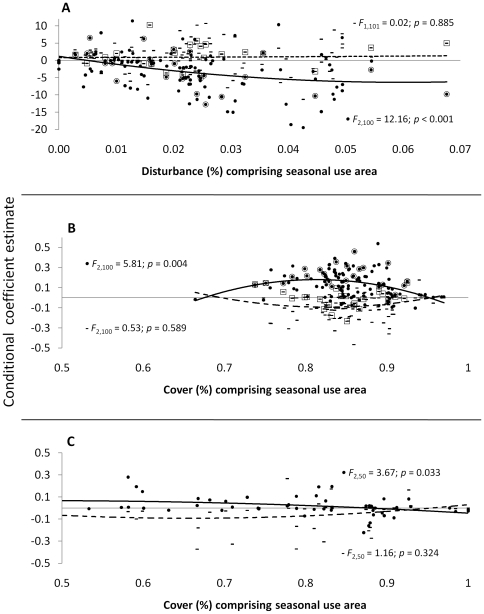
Functional responses in resource selection by female elk. Selection for the human disturbance footprint (a), security cover inside of the gas field (b), and security cover outside of the gas field (c) change as a function of availability. Availability was calculated at the seasonal use area level and conditional coefficients were estimated from generalized linear random-effects resource selection models. Dashes (-) and dashed lines symbolize night-time resource selection, and circles (•) and solid lines symbolize day-time resource selection. Dashes and circles outlined by open circles (○) or boxes (□) indicate conditional estimates for field-observed parturient elk in 2008 and 2009.

Consistent with a functional response in resource selection, elk selected randomly for disturbed areas when disturbance was minimal. As human activity increased (*i.e.*, across individual elk calving season areas, or through time), elk showed stronger avoidance of the industrial development footprint during the day but not at night; this spatiotemporal pattern of avoidance revealed that elk continued to use physically disturbed areas but modified their behavior to avoid human activity (Figure 7a). One way elk avoided human activity in the day was by modifying selection for security cover. In developed areas elk were constrained to select resources near physical disturbance and, during the day, showed a stronger functional response to the proportion of cover than did elk in undeveloped areas (Figure 7b, c).

### Mapping Responses

**Figure 8:**
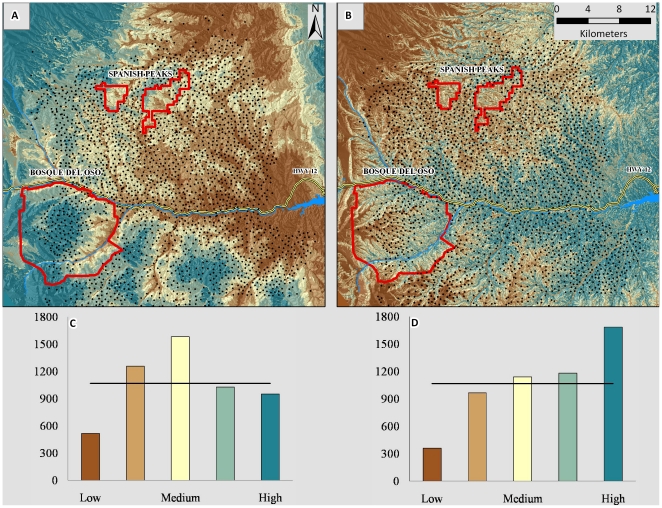
Assigning the relative probability of use throughout the landscape. Comparison of how the relative predicted probability of use was assigned throughout the landscape depending on whether RSF mapping was based on resource selection behavior among female elk that occupied a natural gas field (a) or occupied areas adjacent to but outside of a natural gas field (b). Maps depict day-time probability of use. Wells are depicted as black dots. Borders of wildlife areas managed by the state of Colorado are depicted in red. Probability of use is scaled from low to high with each of 5 ordinal bins representing quantiles of the total number of pixels (30-m resolution) comprising the area. Charts (c, d) display map validation with columns depicting the number of locations from an independent sample of elk that, when plotted on RSF maps (a, b), occurred within each ordinal bin. The black horizontal line depicts expectation if resources were selected at random. The map based on behavior inside of the gas field validated poorly (c; *χ* ^2^ = 577.46, *df* = 4, *p*<0.001) relative to the map based on behavior outside of the gas field (d; *χ* ^2^ = 849.18, *df* = 4, *p*<0.001) with the distribution of validation locations differing significantly from random in both cases. RSF maps (a, b) are based on all sample elk inside and outside of the gas field, respectively; not shown is the RSF map based on elk predicted to be parturient using generalized additive models.

We developed six models (day and night separately for each of three groups): the first was based on all sample elk that occurred inside of the gas field; the second was a subset of the first group for which GAMs predicted females to be parturient; and the third was based on all sample elk that occurred outside of the gas field. We validated day and night models separately (1830 night locations and 3505 day locations) and then summed day and night values within the three groups of interest to estimate overall within-group model performance. For both models based on elk behavior inside of the gas field (Figure 8a), habitat with low predicted probability of use was modeled accurately; however, validation elk used habitat predicted to have high probability of use only as much as would be expected if elk used all predictive classes equally (Figure 8c). The model based on elk behavior outside of the gas field (Figure 8b) validated well with few locations occurring in lowest ranked areas and many locations occurring in highest ranked areas (Figure 8d). The proportion of pixels ranked differently between the model that validated most poorly and the model that validated best was 0.72–0.88 depending on year.

## Discussion

We successfully integrated GAMs, field observation, and random-effects RSF modeling to designate parturiency, describe individual and population-level resource selection, and determine the relative influence of maternal status and human activity on the reliability of spatially explicit models intended to guide conservation of critical ungulate calving resources. We assumed that planning tools such as spatially explicit models would be more reliable if they account for adaptive behavior which, among parturient elk, should be reflected in movement and resource selection [45]. This was the basis for using GAMs to designate parturiency based on movement; our thinking was that ungulate behavior contains information on reproductive status and GAMs would reveal a difference in the shape and relative complexity of the response curve between parturient and non-parturient females. Maternal status was evident in elk movement with our application of GAMs correctly predicting parturiency 93% of the time. We note, however, the GAM approach as presented would require further development if improved sensitivity (reduced false negative rate) is desirable. GAMs have been a standard tool in epidemiologic analyses and have found broad application in ecology for modeling tolerance thresholds and spatial distributions [46], [47], [48], [49]. Using movement data to designate functional groups, seasonality, or behavior in animals has increased in prevalence as GPS-based research has become more frequent [50], [51]. Although we found some potentially important differences in resource selection patterns between parturient and non-parturient elk (see below), behavior in these groups was more similar than expected. Had maternal status been more apparent in shaping resource selection, methods to designate status might have found better application in landscape-level mapping of the relative predicted probability of use. Here, both parturient and non-parturient elk were examined inside the gas field where any behavior distinctly associated with parturiency was obscured by apparent risk-aversive behavior related to human activity during the day time.

At the population-level parturient elk, while avoiding roads and selecting for security cover more strongly than other elk, conformed to a general pattern of avoiding human activity during the day by occupying upland forest or *Q*. *gambelii* thickets, and selecting forage resources in valley bottoms at night regardless of proximity to infrastructure. Trade-offs that structure resource selection in many ungulates, most notably between forage requirements and risk avoidance [6], [52], often are amplified in parturient females through nutritional demands to support body condition, lactation, and neonatal defense [53]. Here, responses to human activity were more apparent than differences in resource selection as a function of parturiency with elk exhibiting a clear spatiotemporal avoidance of human activity at the population-level and modified patterns of selection for environmental features, notably security cover and elevation, in developed areas relative to undeveloped areas. Research has shown that ungulates exhibit avoidance behavior relative to human development, recreation, hunting, and other activities [54]. Modeling resource selection separately during day and night offered insight into whether the physical presence of infrastructure versus the operation and maintenance of such infrastructure (only occurring during the day) was more important in avoidance behavior. Elk inside the gas field used cover and elevation to modulate avoidance of human activity during the day [55] whereas cover and elevation were less influential outside of the gas field suggesting that human activity during the day was the factor to which elk were responding [3]. At night during calving time female elk showed no aversion to infrastructure and selected for areas characterized by valley-bottoms and foraging resources that in many instances were in close proximity to, or directly associated with, disturbances such as well pads and roads [56]. Day time refugia characterized by minimal human disturbance, security cover, and upper slope positions will be critical for maintenance of calving and perhaps other seasonal habitats in multiple-use landscapes.

To our knowledge, fully random-effects RSF models do not appear in the published literature. The random-effects framework provided insight into individual behavior and how elk modified patterns of calving season resource selection relative to development. Individuals that moved between developed and undeveloped areas (Figure 6) were spatially aware and showed marked changes in their behavior that were consistent with the hypothesis that human activity during the day was perceived as a source of risk [6]. An observation that warrants further attention in wildlife-human interaction studies is that heterogeneity among individuals in response to their environment was apparent, and this variation was amplified in developed areas relative to undeveloped areas. Associated with this observation was generally more precise estimation of selection within individuals in developed areas (except selection for elevation). Among-individual variation can comprise the majority of a population's niche width, and only when the within-individual component of total niche width is constrained does between-individual variation become prominent. Of particular relevance here is that trade-offs remain among the most plausible mechanisms for the observation of limited within-individual variation [18]. If ungulates in developed areas must make trade-offs associated with avoiding human activity, particularly during daylight hours, we might expect constrained within-individual variation and thus more heterogeneity among individuals. Human activity functioning to constrain decision-making in ungulates is consistent with the notion that risky environments impose pressures that disallow animals to respond to other features as they otherwise would [57]. This could make it difficult to establish or predict general patterns of resource selection during periods in which ungulates show fidelity to historic ranges, yet human activity rapidly modifies the landscape. In fact, we showed that RSF maps based on day-time resource selection behavior in developed areas had poor predictive accuracy.

The study of behavioral syndromes offers a relevant framework within which to discuss the conservation implications of individual variation in human-wildlife interaction studies [58]. A behavioral syndrome is a suite of correlated behaviors reflecting among-individual consistency across multiple contexts. Within a syndrome, individuals have a behavioral type such as risk-aversion (*i.e.*, more risk-averse versus less risk-averse types; [58]). The notion that individuals can be more or less risk aversive implies a limit to their range of behavioral plasticity. From a conservation perspective, animals exhibiting limited plasticity in environments undergoing rapid change, such as those affected by industrial development, may be less able to adapt. Elk clearly show some ability to adapt to human activity [59]. Nonetheless, if the effect of human activity is a threshold phenomenon [60], we might expect there to exist a limit of physical disturbance corresponding to a limit in the range of behavioral plasticity in ungulates, beyond which redistribution, social, or demographic effects may be observed [61], [62].

Examining only average responses across populations obscures variability among individuals that may have important implications for management or provide new ecological insight. For example, management strategies designed to conserve a resource that is important, on average, to the population may overlook resources that are critical to individuals that comprise a smaller demographic segment that functions disproportionately in population persistence. It has been stated “information on individual resource use is necessary if we are to make the transition from phenomenological models of population dynamics to mechanistic models in which the dynamics of a population are predicted from the properties of its components” [18]. Conditional estimates also provide information on why marginal estimates may be counter-intuitive, or how individuals assemble to comprise the marginal estimate. For example, the marginal estimate of selection for road density in our study indicated that elk selected for higher road density in 3 of the 4 years comprising the study period (Figure 5). This observation is counter to most research on roads and ungulates [63], [64]. Conditional estimates revealed that ∼1/3 of elk showed a positive association with higher road density but just as many avoided high road density; this observation sets the stage for examining potential links between a particular behavioral strategy such as road avoidance and demographic responses. The increase in the within-year proportion of elk occupying areas with higher road density from 2007 to 2009 (Figure 5d), concurrent with an increase in new road development, suggests that elk maintained relatively consistent calving season use areas during the study period, but modified their behavior as they became increasingly constrained to select resources in proximity to development. Such modification was consistent with a functional response in resource selection; elk response to human activity changed as a function of availability and with time of day. During the day when humans are active elk showed avoidance behavior that strengthened with increasing disturbance. This response spilled over to elk selection for security cover which appeared to have high importance to elk inside of the gas field as a day time refugium. Outside of the gas field elk showed relatively weak day time selection for cover and, as might be expected, selection weakened as the proportion of cover within the seasonal use area increased (*sensu* [40], Figure 7). These observations are consistent with the hypothesis that ungulates face a trade-off that is mediated by human activity in multiple-use landscapes – that is, the strength of the trade-off varies in direct relation to resource availability which is driven by human activity [41].

Spatially explicit models of relative predicted probability of use validated poorly when they were based on resource selection behavior inside of the gas field. The model based on behavior outside of the gas field validated well. To our knowledge, this treatment/control concept is a novel approach in RSF-based conservation planning. Typically, quantifying relative predicted probability of use in human-modified areas is based on animal behavior in those areas. This approach has been used effectively; it has been shown that predictive maps based on mule deer (*Odocoileus hemionus*) behavior in human-modified areas validated well [3]. In their study [3], variation among individuals was present but limited relative to our findings. We suggest that ungulates in developed areas often respond to human activity in situation-specific ways. In our study, the local attributes of human activity varied in space and time within the seasonal use area of each elk making it difficult to generalize across the population. Situation-specific responses, including individual variation in the strength of selection or avoidance response, induced heterogeneity which complicated the application of models based on such behavior for conservation planning.

Protecting unmodified habitat in multiple-use landscapes typically is not an option [65]; therefore, measures to account for or reconcile changes in animal behavior across gradients of human-modification might be considered. If conservation objectives include establishing zones within such landscapes intended to function as refugia from human activity and promote long-term population persistence, information on resource selection patterns in existing refugia would be expected to provide valuable guidance in prioritizing the creation of new zones in modified landscapes. Establishing refugia based on resource selection patterns that reflect responses to human activity risks uncertainty in the performance of such refugia once human disturbance pressure is released allowing animals to respond to features as they otherwise would have [57]. In our study, models based on resource selection patterns that reflected responses to human activity classified the relative probability of use differently from the models based on behavior in the absence of industrial development throughout extensive portions of the landscape, potentially resulting in large errors in where conservation action would have its greatest impact. In population response research it is always desirable to include spatial and temporal controls (before-after, control-impact design), and demographic responses. Availability of such components is uncommon in large-scale wildlife-human interaction studies. In such situations the approach we describe, including efforts to account for possible adaptive behavior among reproductive groups, attention to the treatment/control concept, and a random-effects modeling framework, should have general application in human-wildlife interaction research particularly among species that inhabit places where human activity is intense, or among special status species for which little information on resource needs exists.

## References

[1] Bowyer RT, Van Ballenberghe V, Kie JG, Maier JK (1999). Birth-site selection by Alaskan moose: maternal strategies for coping with a risky environment.. Journal of Mammalogy.

[2] White PJ, Davis TL, Barnowe-Meyer KK, Crabtree RL, Garrott RA (2006). Partial migration and philopatry of Yellowstone pronghorn.. Biological Conservation.

[3] Sawyer H, Kauffman MJ, Nielsen RM (2009). Influence of well pad activity on winter habitat selection patterns of mule deer.. Journal of Wildlife Management.

[4] Hebblewhite M, Merrill E (2008). Modelling wildlife-human relationships for social species with mixed-effects resource selection models.. Journal of Applied Ecology.

[5] Fortin D, Courtois R, Etcheverry P, Dussault C, Gingras A (2008). Winter selection of landscapes by woodland caribou: behavioral response to geographical gradients in habitat attributes.. Journal of Applied Ecology.

[6] Frid A, Dill L (2002). Human-caused disturbance stimuli as a form of predation risk.. Conservation Ecology.

[7] Beale CM, Monaghan P (2004). Human disturbance: people as predation-free predators?. Journal of Applied Ecology.

[8] Creel S, Christianson D, Liley S, Winnie JA (2007). Predation risk affects reproductive physiology and demography of elk.. Science.

[9] Manly BFJ, McDonald LL, Thomas DL, McDonald TL, Erickson WP (2002). Resource selection by animals: statistical design and analysis for field studies..

[10] Kittle AM, Fryxell JM, Desy GE, Hamr J (2008). The scale-dependent impact of wolf predation risk on resource selection by three sympatric ungulates.. Oecologia.

[11] Fortin D, Fortin ME, Beyer HL, Duchesne T, Courant S (2009). Group-size-mediated habitat selection and group fusion-fission dynamics of bison under predation risk.. Ecology.

[12] Singer FJ, Harting A, Symonds KK, Coughenour MB (1997). Density dependence, compensation, and environmental effects on calf mortality in Yellowstone National Park.. Journal of Wildlife Management.

[13] Phillips GE, Alldredge AW (2000). Reproductive success of elk following disturbance by humans during calving season.. Journal of Wildlife Management.

[14] Schaefer JA, Bergman CM, Luttich SN (2000). Site fidelity of female caribou at multiple spatial scales.. Landscape Ecology.

[15] Bernardo J (1996). Maternal effects in animal ecology.. Am Zool.

[16] Mousseau TA, Fox CW (1998). The adaptive significance of maternal effects.. Trends in Ecology and Evolution.

[17] Blount JD, Surai PF, Nager RG, Houston DC, Møller AP (2002). Carotenoids and egg quality in the lesser black-backed gull *Larus fuscus*: a supplemental feeding study of maternal effects.. Proceedings of the Royal Society B.

[18] Bolnick DI, Svanbäck R, Fordyce JA, Yang LH, Davis JM (2003). The ecology of individuals: incidence and implications of individual specialization.. The American Naturalist.

[19] Austin D, Bowen WD, McMillan JI (2004). Intraspecific variation in movement patterns: modeling individual behavior in a large marine predator.. Oikos.

[20] Thompson MJ, Henderson RE (1998). Elk habituation as a credibility challenge for wildlife professionals.. Wildlife Society Bulletin.

[21] Estes JA, Riedman ML, Staedler MM, Tinker MT, Lyon BE (2003). Individual variation in prey selection by sea otters: patterns, causes, and implications.. Journal of Animal Ecology.

[22] Gillespie RG, Caraco T (1987). Risk-sensitive foraging strategies of two spider populations.. Ecology.

[23] Western Regional Climate Center (2010). http://www.wrcc.dri.edu/coopmap/.

[24] Hemborg HT (1998). Spanish Peak Field, Las Animas County, Colorado: Geologic setting and early development of a coalbed methane reservoir in the central Raton basin.. Colorado Geological Survey Department of Natural Resources, Resources Series.

[25] Vitt A (2007). Trinchera Data Analysis Unit E-33, Game Management Units 83, 85, 140, 851, Elk Management Plan..

[26] Quimby DC, Gaab JE (1957). Mandibular dentition as an age indicator in Rocky Mountain elk.. Journal of Wildlife Management.

[27] Hastie TJ, Tibshirani RJ (1990). Generalized additive models..

[28] Guisan A, Edwards TC, Hastie T (2002). Generalized linear and generalized additive models in studies of species distributions: setting the scene.. Ecological Modelling.

[29] Beck N, Jackman S (1997). Getting the mean right is a good thing: generalized additive models..

[30] Fewster RM, Buckland ST, Siriwardena GM, Baillie SR, Wilson JD (2000). Analysis of population trends for farmland birds using generalized additive models.. Ecology.

[31] Clutton-Brock TH, Guinness FE (1975). Behaviour of red deer (*Cervus elaphus* L.) at calving time.. Behaviour.

[32] Cowie GM, Moore GH, Fisher MW, Taylor MJ (1985). Calving behavior of farmed red deer.. Proceedings of a Deer Course for Veterinarians 2:1.

[33] Hudson RJ, Haigh JC, Toweill DE, Thomas JW (2002). Physical and physiological adaptations.. North American elk: ecology and management.

[34] Vore JM, Schmidt EM (2001). Movements of female elk during the calving season in northwest Montana.. Wildlife Society Bulletin.

[35] Poole KG, Serrouya R, Stuart-Smith K (2007). Moose calving strategies in interior montane ecosystems.. Journal of Mammalogy.

[36] Long RA, Kie JG, Bowyer RT, Hurley MA (2009). Resource selection and movements by female mule deer *Odocoileus hemionus*: effects of reproductive stage.. Wildlife Biology.

[37] Noyes JH, Sasser RG, Johnson BK, Byrant LD, Alexander B (1997). Accuracy of pregnancy detection by serum protein (PSPB) in elk.. Wildlife Society Bulletin.

[38] Johnson DE (1951). Biology of the elk calf, *Cervus elaphus nelsoni*.. Journal of Wildlife Management.

[39] Gillies CS, Hebblewhite M, Nielsen SE, Krawchuk MA, Aldridge C (2006). Application of random effects to the study of resource selection by animals.. Journal of Animal Ecology.

[40] Mysterud A, Ims RA (1998). Functional responses in habitat use: availability influences relative use in trade-off situations.. Ecology.

[41] Godvik IMR, Loe LE, Vik JO, Veiberg V, Langvatn R (2009). Temporal scales, trade-offs, and functional responses in red deer habitat selection.. Ecology.

[42] Sawyer H, Nielson RM, Lindzey F, McDonald LL (2006). Winter habitat selection of mule deer before and during development of a natural gas field.. Journal of Wildlife Management.

[43] Marzluff JM, Millspaugh JJ, Hurvitz P, Handcock MS (2004). Relating resources to a probabilistic measure of space use: forest fragments and Steller's jays.. Ecology.

[44] Thomas DL, Johnson D, Griffith B (2006). A Bayesian random effects discrete-choice model for resource selection: population-level selection inference.. Journal of Wildlife Management.

[45] Geist V, Toweill DE, Thomas JW (2002). Adaptive behavioral strategies.. North American elk: ecology and management.

[46] Dominici F, McDermott A, Zeger SL, Samet JM (2002). On the use of generalized additive models in time-series studies of air pollution and health.. American Journal of Epidemiology.

[47] Yuan LL (2004). Assigning macroinvertebrate tolerance classifications using generalized additive models.. Freshwater Biology.

[48] Potvin MJ, Drummer TD, Vucetich JA, Beyer DE, Peterson RO (2005). Monitoring and habitat analysis for wolves in upper Michigan.. Journal of Wildlife Management.

[49] Winter AG, Swartzman GL (2006). Interannual changes in distribution of age-0 walleye Pollock near the Pribilof Islands, Alaska, with reference to the prediction of Pollock year-class strength.. Journal of Marine Science.

[50] Franke A, Caelli T, Kuzyk G, Hudson RJ (2006). Prediction of wolf (*Canis lupus*) kill-sites using hidden Markov models.. Ecological Modelling.

[51] Vander Wal E, Rodgers AR (2009). Designating seasonality using rate of movement.. Journal of Wildlife Management.

[52] Bowyer RT, Kie JG, Van Ballenberghe V (1998). Habitat selection by neonatal black-tailed deer: climate, forage, or risk of predation?. Journal of Mammalogy.

[53] Keech MA, Bowyer RT, Ver Hoef JM, Boertje RD, Dale BW (2000). Life-history consequences of maternal condition in Alaskan moose.. Journal of Wildlife Management.

[54] Morrison JR, de Vergie WJ, Alldredge AW, Byrne AE, Andree WW (1995). The effects of ski area expansion on elk.. Wildlife Society Bulletin.

[55] Edge WD, Marcum CL (1991). Topography ameliorates the effects of roads and human disturbance on elk. In: Christensen AG, Lyon LJ, Lonner TN, editors. Proceedings of a symposium on elk vulnerability. Bozeman: Montana State University.. pp.

[56] Anderson DP, Turner MG, Forester JD, Zhu J, Boyce MS (2005). Scale-dependent summer resource selection by reintroduced elk in Wisconsin, USA.. Journal of Wildlife Management.

[57] Winnie J, Christianson D, Creel S, Maxwell B (2006). Elk decision-making is simplified in the presence of wolves.. Behavioral Ecology and Sociobiology.

[58] Sih A, Bell A, Johnson JC (2004). Behavioral syndromes: and ecological and evolutionary overview.. Trends in Ecology and Evolution.

[59] Edge WD, Marcum CL (1985). Movements of elk in relation to logging disturbances.. Journal of Wildlife Management.

[60] Harju SM, Dzialak MR, Taylor RC, Hayden-Wing LD, Winstead JB (2010). Thresholds and time lags in the effects of energy development on greater sage-grouse populations.. Journal of Wildlife Management.

[61] Cameron RD, Reed DJ, Dau JR, Smith WT (1992). Redistribution of calving caribou in response to oil field development on the Arctic slope of Alaska.. Arctic.

[62] Manor R, Saltz D (2003). Impact of human nuisance disturbance on vigilance and group size of a social ungulate.. Ecological Applications.

[63] Rowland MM, Wisdom MJ, Johnson BK, Kie JG (2000). Elk distribution and modeling in relation to roads.. Journal of Wildlife Management.

[64] Friar JL, Merrill EH, Beyer HL, Morales JM (2008). Thresholds in landscape connectivity and mortality risks in response to growing road networks.. Journal of Applied Ecology.

[65] Moilanen A, Franco AMA, Early RI, Fox R, Wintle B, Thomas CD (2005). Prioritizing multiple-use landscapes for conservation: methods for large multi-species planning problems.. Proceedings of The Royal Society B.

